# Supplementary material for: Semaphorin-Plexin signaling influences early ventral telencephalic development and thalamocortical axon guidance
Source: Neural Dev. 2017 Apr 24;12:6. doi: 10.1186/s13064-017-0083-4 (PMC5402653; doi:10.1186/s13064-017-0083-4)
Supplement: Additional file 1: — Supplemental Material. (DOCX 7186 kb) [file 13064_2017_83_MOESM1_ESM.docx]

**Supplemental Material**

**Semaphorin-Plexin signaling influences early ventral telencephalic development and thalamocortical axon guidance**Manuela D. Mitsogiannis, Graham E. Little, Kevin J. Mitchell

**Table S1** **Primers employed to genotype embryos/mice from the *Sema6a* and *Plxna2*;*Plxna4* lines, and relative PCR product sizes.**

| **Target gene** | **Primers** | **PCR products** |
| --- | --- | --- |
| *Sema6a* | Forward:  5’-GAGATGCACAGCTAACTTCTGGTG-3’  Reverse (wild-type allele):  5’-TTGAAGCCTGCTCTTAGTGGCTCC-3’  Reverse (mutant allele):  5’-GCTACCGGCTAAAACTTGAGACCT-3’ | Wild-type allele:  1.43 Kb  Mutant allele:  0.99 Kb |
| *Plxna2* | Forward (wild-type allele):  5’-GCTGGAACCATGTGAGAGCTGATC-3’  Forward (mutant allele):  5’-GGTCATCTAGTCGCAGGAGCTTGC-3’  Reverse:  5’-TACCCGTGATATTGCTGAAGAGCTTGG-3’ | wild-type allele:  0.51 Kb mutant allele:  0.93 Kb |
| *Plxna4* | Forward (wild-type allele):  5’-CCATGCTCTCCTTCAGCCTGCTCT-3’  Forward (mutant allele):  5’-GCTAAAGCGCATGCTCCAGACTGC-3’  Reverse:  5’-CTTCAGCACTGGCTGCTGTCATCT-3’ | wild-type allele:  0.68 Kb  mutant allele:  0.43 Kb |

Primer sequences and product sizes for *Plxna2* and *Plxna4* alleles were obtained respectively from Suto et al. [1] and Yaron et al. [2].

**Table S2** **Details of riboprobes used for *in situ* hybridization experiments.**

| **Probe** | **Obtained from** | **Size (bps)** | **Linearization** | **Product** |
| --- | --- | --- | --- | --- |
| SD89 (Sema6A) | Kevin J. Mitchell | 280 | XhoI | as-Sema6A |
| PlxnA2 | Alain Chédotal | 315 | XhoI | as-PlxnA2 |
| PlxnA4 | Alain Chédotal | 1260 | EcoRV | as-PlxnA4 |

**
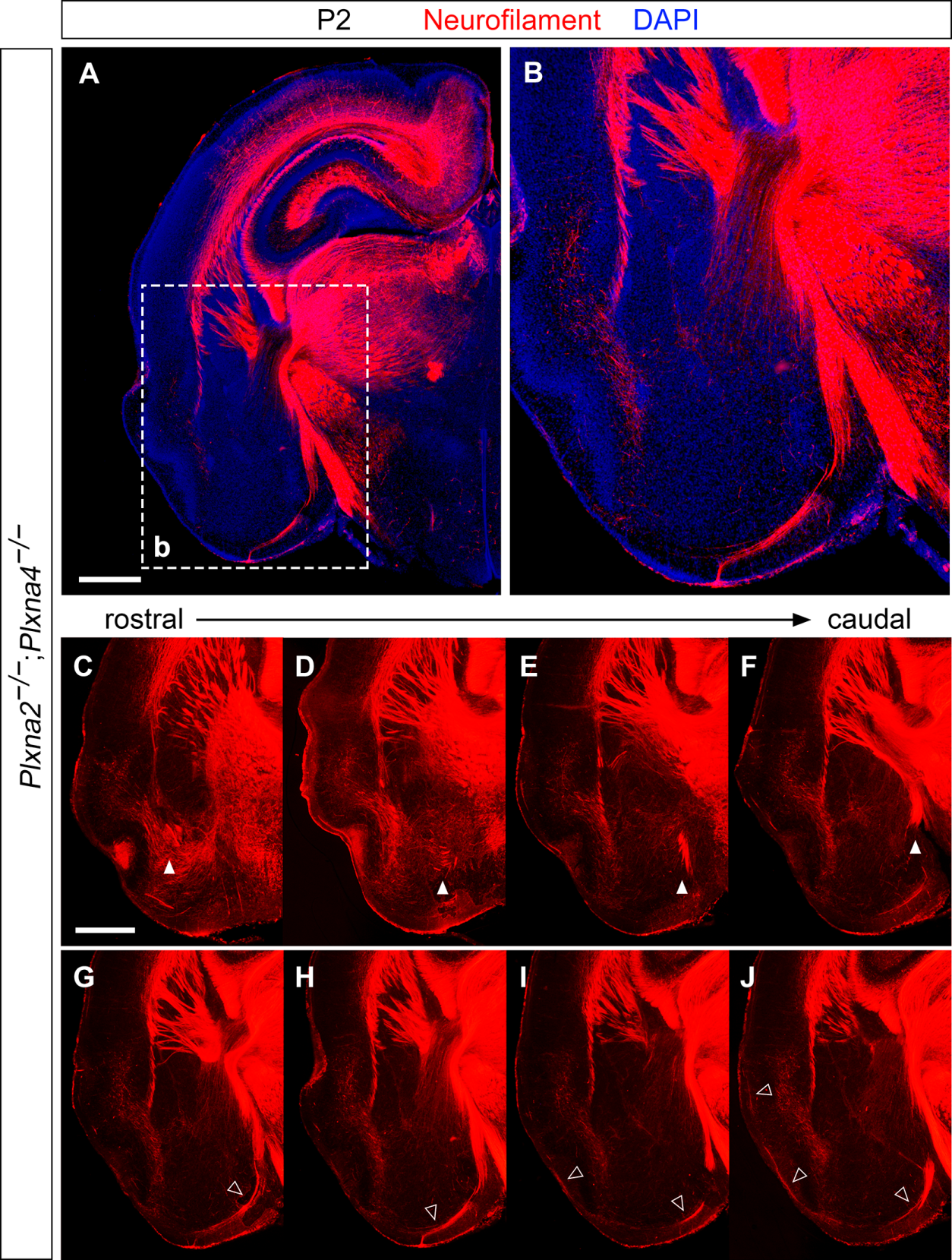
**

**Figure S1 Immunohistochemical analysis of thalamocortical tract defects in a *Plxna2*;*Plxna4* double mutant P2 brain.** Neurofilament immunostaining (red) reveals the presence of misrouted TCAs in the vTel of *Plxna2*;*Plxna4* double mutants (A, B). This TCA phenotype is markedly similar to that observed in *Sema6a* homozygous mutants; at more rostral levels (C–F), TCAs extend mainly along the external capsule (filled arrowheads), while at caudal levels (G–J) TCAs mostly follow a ventral route in the vTel, then proceed laterally across the telencephalon following a superficial pathway (empty arrowheads).

Scale: A, 500 μm; B, 200 μm; C–J, 500 μm.

**
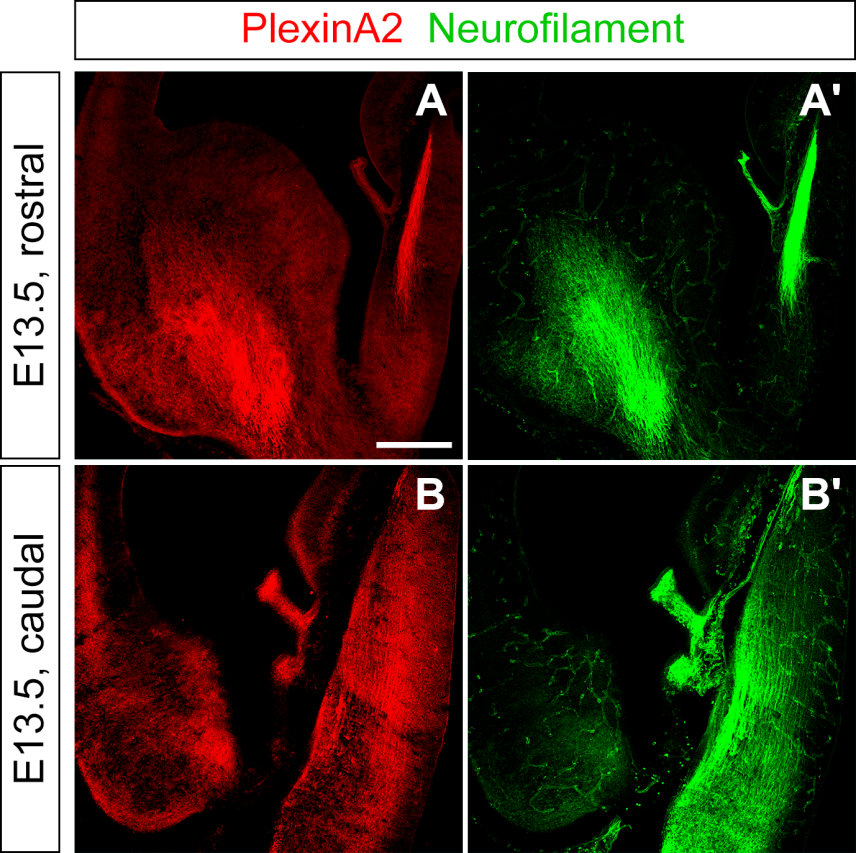
**

**Figure S2** **Expression of PlxnA2 on thalamic neurons and TCAs during axonal growth into the subpallium.** Double immunohistochemistry for PlxnA2 (red) and neurofilament (green) on wild-type E13.5 coronal brain sections reveals that expression of PlxnA2 is localized in medial thalamic neural populations (B–B’). The protein is additionally present on TCAs; high expression can also be observed in a restricted domain immediately dorsal to the IC, likely correspondent to the corridor (A–A’).

Scale: 250 μm.


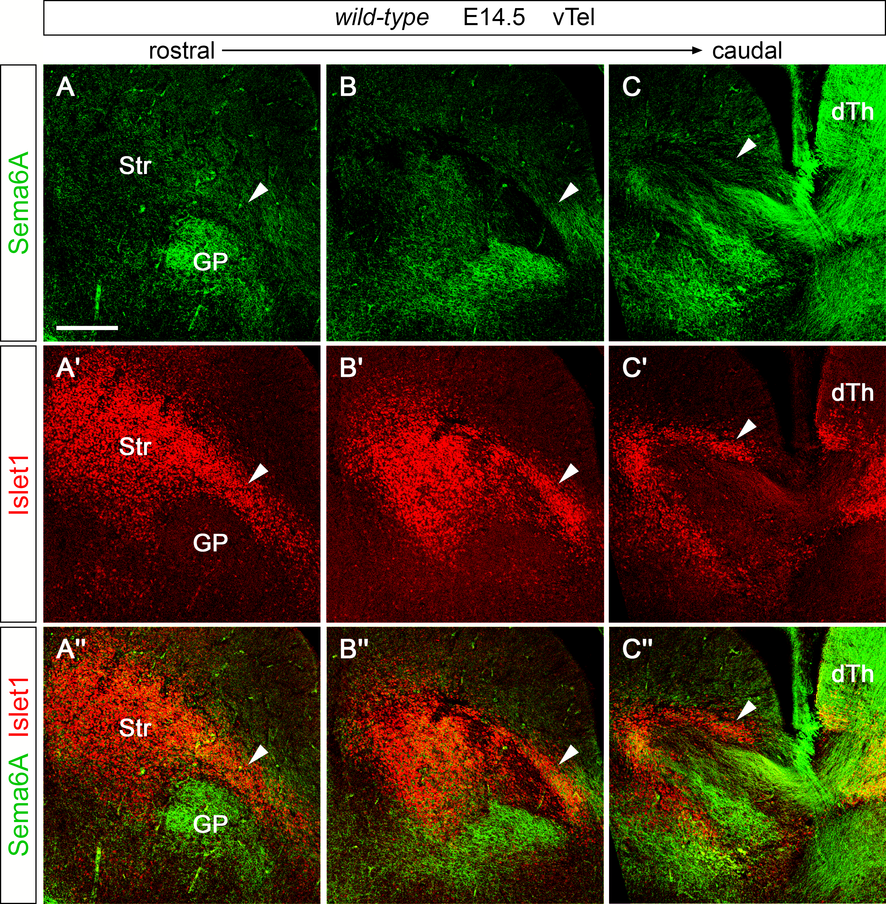


**Figure S3** **Subpallial Sema6A–Islet1 co-expression domains throughout the rostro-caudal axis at E14.5.** Double immunohistochemistry for Sema6A (green, A−C) and Islet1 (red, A’−C’) on coronal E14.5 wild-type brain sections reveals moderate expression of Sema6A on corridor cells (white arrowheads) throughout the rostro-caudal axis (A’’−B’’). Sema6A can additionally be observed at high levels in the globus pallidus (GP) (A’’, B’’), in the dTh and on TCAs (C’’), and caudally in ventral surface zones of the subpallium (C’’). Sema6A appears to be expressed also in other tracts extending into the IC (possibly the nigrostriatal / striatonigral pathways, that traverse the IC ventrally to the thalamocortical bundle [3]) (C’’).

Scale: 200 μm.


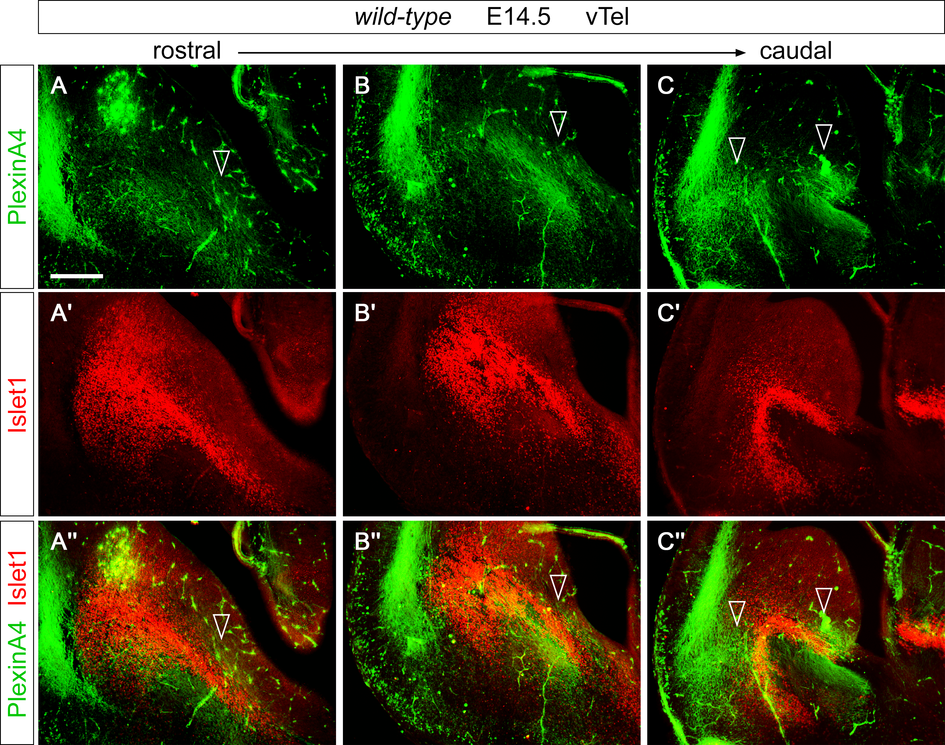


**Figure S4** **Subpallial PlxnA4–Islet1 co-expression domains throughout the rostro-caudal axis at E14.5.** Double immunohistochemistry for PlxnA4 (green, A−C) and Islet1 (red, A’−C’) on coronal E14.5 wild-type brain sections reveals that some corridor cells, in particular those located in its caudal-most portions (C’’), express PlxnA4 (A’’−C’’). The protein is additionally observed in a band dorsal to the corridor region, which can be observed throughout the rostro-caudal axis, and a discrete area lateral to it, localized more caudally in the vTel (empty arrowheads). Moderate expression can be moreover detected in the globus pallidus (A’’, B’’).

Scale: 250 μm.


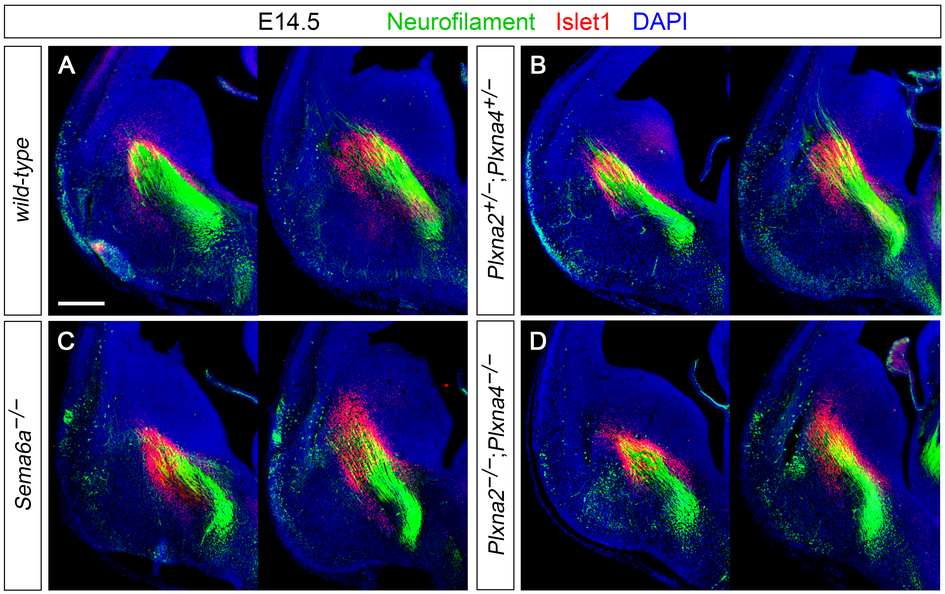


**Figure S5** **Normal overall expression of Islet1 in the vTel of *Sema6a* mutants and *Plxna2;Plxna4* double mutants at E14.5.** Double immunohistochemistry for neurofilament (green) and Islet1 (red) on E14.5 coronal brain sections reveals similar patterns of expression between wild-type (A) and *Plxna2*^+/−^;*Plxna4*^+/−^ (B), *Sema6a*^−/−^ (C), *Plxna2*^−/−^;*Plxna4*^−/−^ (D) mouse brains (each panel represents data from a distinct animal). The corridor marker is detectable both in a discrete band dorsal to the site of TCAs extension, between the MGE subventricular zone and the globus pallidus, and across the striatum; no expression is found in MGE-derived territories.

Scale: 250 μm.

**
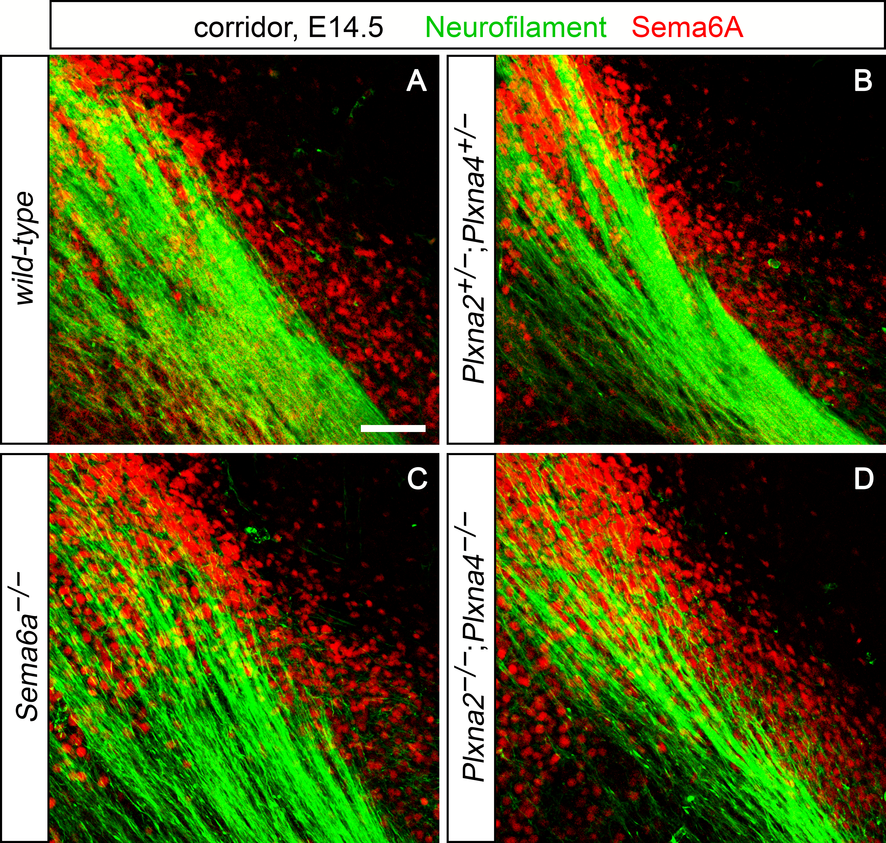
**

**Figure S6  Comparison of the corridor area between wild-type, *Sema6a* mutant and *Plxna2;Plxna4* double mutant E14.5 brains.** All panels represent coronal brain sections taken at the same rostro-caudal position. Double immunohistochemistry for neurofilament (green) and Islet1 (red) shows the similar distribution and density of Islet1-positive cells at the level of the TCA-permissive corridor in wild-type (A), *Plxna2*^+/−^;*Plxna4*^+/−^ (B), *Sema6a*^−/−^ (C) and *Plxna2*^−/−^;*Plxna4*^−/−^ (D) mouse brains.

Scale: 50 μm.


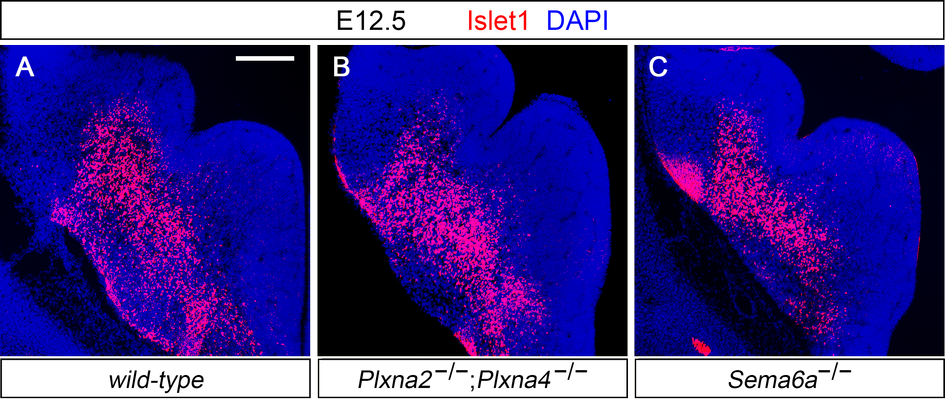


**Figure S7** **Normal migration of Islet1+ corridor cells at E12.5 in *Sema6a* mutants and *Plxna2;Plxna4* double mutant brains.** Islet1+ corridor cells (red) appear to migrate in a wild-type-comparable manner (A) in the vTel in both *Plxna2*^−/−^;*Plxna4*^−/−^ (B) and *Sema6a*^−/−^ (C) mutant brains, forming a corridor-like structure in MGE-derived territories as TCAs start crossing the DTB to invade the subpallium.

Scale: 200 μm.

**References:**

1. Suto, F., et al., *Interactions between plexin-A2, plexin-A4, and semaphorin 6A control lamina-restricted projection of hippocampal mossy fibers.* Neuron, 2007. **53**(4): p. 535-547.

2. Yaron, A., et al., *Differential requirement for Plexin-A3 and -A4 in mediating responses of sensory and sympathetic neurons to distinct class 3 Semaphorins.* Neuron, 2005. **45**(4): p. 513-523.

3. Uemura, M., et al., *OL-Protocadherin is essential for growth of striatal axons and thalamocortical projections.* Nature Neuroscience, 2007. **10**(9): p. 1151-1159.
